# Clinical Efficacy of the GentleWave® System Compared to Conventional Irrigation Techniques: A Systematic Review

**DOI:** 10.7759/cureus.117411

**Published:** 2026-10-03

**Authors:** Tejas Nemade, Ashish K Jain, Rahul Rao, Sanpreet S Sachdev, Amit Patil, Ashwini Bharitkar

**Affiliations:** 1 Conservative Dentistry and Endodontics, Bharati Vidyapeeth (Deemed to be University) Dental College and Hospital, Navi Mumbai, IND; 2 Oral Pathology and Microbiology, Bharati Vidyapeeth (Deemed to be University) Dental College and Hospital, Navi Mumbai, IND

**Keywords:** irrigation system, minimally invasive instrumentation, negative-pressure fluidics, post-operative discomfort, root-canal irrigation, ultrasonic activation

## Abstract

Effective root canal disinfection depends on irrigation, but newer technologies require careful clinical evaluation. The GentleWave® system (Sonendo Inc., Laguna Hills, USA) uses multisonic energy and fluid dynamics and has been proposed to improve debridement while limiting canal enlargement. This systematic review evaluated GentleWave-assisted root canal treatment for postoperative pain, clinical and radiographic healing, and clinically reported disinfection outcomes. The review was registered in PROSPERO (CRD420250648962) and conducted according to Preferred Reporting Items for Systematic Reviews and Meta-Analyses (PRISMA) 2020. PubMed/MEDLINE, Embase, Scopus, Web of Science, Cochrane Central Register of Controlled Trials (CENTRAL), Google Scholar, and EBSCOhost databases were searched from inception to October 21, 2025. Human clinical studies reporting at least one prespecified outcome were eligible. Owing to clinical and methodological heterogeneity, findings were synthesized descriptively. Five publications were included: two randomized controlled trials and three reports from prospective noncomparative cohorts. A pooled participant total was not calculated because two publications represented different follow-up periods from the same cohort and further overlap could not be excluded. Postoperative pain was frequently reported early in the randomized trials, but mean intensity was generally low to mild and decreased over time. Neither trial demonstrated a statistically significant between-group difference during the first postoperative week. The noncomparative publications reported combined clinical and radiographic healing proportions exceeding 97% at six to 12 months. No eligible study directly evaluated intracanal disinfection clinically. Low-certainty randomized evidence therefore did not demonstrate a clear short-term pain advantage of GentleWave over conventional syringe irrigation with ultrasonic activation. The high healing proportions were derived from noncomparative, potentially overlapping evidence of very low certainty. Adequately powered comparative studies with longer follow-up are needed.

## Introduction and background

Root canal treatment is a fundamental component of contemporary endodontic care and is intended to preserve teeth affected by irreversible pulpitis, pulpal necrosis, or apical periodontitis [1]. Its long-term success depends on reducing the intracanal microbial load and preventing reinfection through effective obturation and coronal sealing [2]. However, mechanical instrumentation cannot contact the entire root canal surface or consistently reach anatomical complexities such as fins, lateral canals, isthmuses, and apical deltas. Isthmuses are narrow connections between canals, whereas apical deltas are networks of small canal branches near the root apex. Chemical irrigation is therefore essential for flushing debris, dissolving organic tissue, disrupting biofilms, and extending debridement beyond areas directly contacted by instruments [3-5].

Complete debridement remains challenging because complex anatomical regions may retain tissue remnants and microorganisms after apparently adequate instrumentation [3,6]. Although uninstrumented surfaces may provide sites for microbial persistence, their presence does not necessarily result in clinical failure because treatment outcomes are influenced by several interacting microbial, anatomical, procedural, and host-related factors [6,7]. Instrumentation also produces a smear layer, consisting of organic and inorganic debris deposited on canal walls. This layer may occlude dentinal tubules, which are microscopic channels within dentin, and may limit the penetration of irrigants and antimicrobial agents [8]. The ability of microorganisms, including *Enterococcus faecalis*, to persist within protected areas further supports the need for effective irrigant delivery and exchange throughout the canal system [9,10].

Conventional positive-pressure irrigation commonly involves delivering sodium hypochlorite and ethylenediaminetetraacetic acid (EDTA) through a syringe and needle [11]. Its effectiveness may be limited by inadequate irrigant replacement near the apical terminus, restricted distribution into lateral anatomical spaces, and the risk of irrigant extrusion beyond the root apex [11,12]. Several adjunctive approaches - including passive ultrasonic irrigation, sonic activation, manual dynamic agitation, and negative-pressure irrigation - have therefore been developed to improve irrigant movement and debris removal [12,13]. Negative-pressure systems draw irrigant toward the apical portion of the canal through suction rather than expressing it apically under positive pressure. In this context, conventional irrigation may include syringe delivery alone or syringe irrigation combined with an activation method. Interest in these approaches has increased alongside minimally invasive endodontic concepts, which aim to preserve dentin but may restrict irrigant delivery when conservative access cavities and smaller canal preparations are used [14].

The GentleWave® system (Sonendo Inc., Laguna Hills, USA) has been introduced as an irrigation technology intended to improve fluid exchange and cleaning within minimally prepared root canals [15]. The system delivers treatment fluids into the pulp chamber and uses suction-assisted evacuation under negative pressure. It also generates a broad range of acoustic frequencies, described as multisonic energy, which is proposed to promote irrigant movement into complex canal anatomy and dentinal tubules [15,16]. These characteristics represent proposed mechanisms and should not be interpreted as established clinical advantages. Laboratory investigations have reported effects on microbial components and contaminants within experimentally infected root canals [17,18]; however, mechanistic plausibility and laboratory performance do not necessarily translate into superior patient-centered or long-term clinical outcomes.

The clinical benefit of GentleWave relative to established irrigation approaches therefore requires careful evaluation. In particular, it remains important to determine whether its proposed hydrodynamic and acoustic mechanisms result in clinically meaningful differences in postoperative symptoms, clinical and radiographic healing, or clinically measured disinfection outcomes. Accordingly, the present systematic review aimed to evaluate the clinical efficacy of GentleWave-assisted root canal treatment compared with conventional irrigation techniques, with emphasis on postoperative pain, clinical and radiographic healing, and clinically reported disinfection-related outcomes.

## Review

Methodology

Protocol Registration and Reporting

The systematic review protocol was registered in the International Prospective Register of Systematic Reviews (PROSPERO; CRD420250648962). The review was conducted and reported in accordance with the Preferred Reporting Items for Systematic Reviews and Meta-Analyses (PRISMA) 2020 guidelines [19].

Eligibility Criteria

Studies were selected using predefined inclusion and exclusion criteria structured according to the PICOS (Population, Intervention, Comparator, Outcomes, Study design) framework (Table 1). Eligible participants were human subjects undergoing primary nonsurgical root canal treatment in permanent teeth, with an identifiable baseline pulpal and/or periapical diagnosis. Studies including both primary treatment and retreatment were eligible only when data for primary root canal treatment could be extracted separately.

**Table 1:** Inclusion and exclusion criteria based on the PICOS framework PICOS: Population, Intervention, Comparator, Outcomes, Study design

Criterion	Inclusion criteria	Exclusion criteria
Population	Human participants undergoing primary nonsurgical root canal treatment in permanent teeth, with baseline pulpal and/or periapical diagnoses clearly reported	Primary teeth; endodontic retreatment; surgical endodontic treatment; studies without an identifiable baseline clinical diagnosis
Intervention	Irrigation using the commercial GentleWave® System (Sonendo Inc., Laguna Hills, USA), typically incorporating sodium hypochlorite and ethylenediaminetetraacetic acid	Studies not evaluating GentleWave or using experimental or substantially modified systems not representative of the commercial clinical protocol
Comparator	Conventional syringe irrigation alone or combined with ultrasonic, sonic, or negative-pressure activation when a comparator was included; prospective single-arm clinical studies were eligible for healing outcomes	Comparator protocols insufficiently described for interpretation or studies in which GentleWave-specific outcomes could not be isolated
Outcomes	At least one prespecified outcome: postoperative pain; clinical or radiographic healing/success; bacterial reduction; smear layer removal; or irrigant penetration into dentinal tubules	Studies reporting none of the prespecified outcomes or providing no extractable outcome data
Study design	Randomized controlled trials, controlled clinical trials, and prospective cohort studies	In vitro or ex vivo studies, animal studies, case reports, case series, retrospective studies, narrative reviews, and systematic reviews
Publication format	Full-text peer-reviewed articles and eligible academic theses	Conference abstracts, editorials, commentaries, protocols, and reports for which the full text was unavailable
Language	English-language reports	Reports published in languages other than English
Publication period	No restriction on publication year; reports available up to the final search in October 2025	Reports published after the final search date

The intervention of interest was irrigation using the commercial GentleWave system, generally incorporating sodium hypochlorite and ethylenediaminetetraacetic acid (EDTA). For comparative studies, eligible comparators included conventional positive-pressure syringe irrigation alone or syringe irrigation combined with ultrasonic, sonic, or negative-pressure activation, provided that the comparator technique and irrigant protocol were adequately described. Prospective single-arm clinical studies were also eligible for the secondary descriptive assessment of postoperative pain and healing outcomes.

The primary outcome was postoperative pain. Secondary outcomes included mechanical allodynia, clinical or radiographic healing or success, bacterial reduction, smear-layer removal, and irrigant penetration into dentinal tubules. Disinfection-related outcomes were eligible only when assessed as part of a human clinical investigation. Randomized controlled trials, controlled clinical trials, prospective comparative cohort studies, and prospective single-arm clinical studies with predefined enrollment and longitudinal follow-up were eligible. In vitro and ex vivo studies, animal studies, retrospective studies, isolated case reports, anecdotal case series without prospective follow-up, conference abstracts, protocols, editorials, commentaries, and narrative or systematic reviews were excluded.

Only English-language full-text reports, including peer-reviewed articles and eligible academic theses or dissertations, were considered. No restriction was applied according to the year of publication. Reports for which the complete text could not be obtained were excluded because their eligibility and outcome data could not be adequately verified. Multiple publications arising from the same or a potentially overlapping participant cohort were treated as separate reports of a related study population rather than as independent studies.

Information Sources and Search Strategy

A comprehensive electronic search was conducted in PubMed/MEDLINE, Scopus, Embase, Web of Science Core Collection, and the Cochrane Central Register of Controlled Trials (CENTRAL). Supplementary searches were conducted through Google Scholar and EBSCOhost, and the first 200 records sorted according to relevance were taken from the search engine results. The EBSCOhost databases searched comprised MEDLINE, MEDLINE Ultimate, MEDLINE with Full Text, CINAHL, CINAHL Ultimate, CINAHL Plus, Biotechnology Source, and Biomedical Reference Collection. All databases and supplementary sources were searched from inception, and the final searches were conducted on October 21, 2025.

Search terms and controlled vocabulary, where available, were combined to identify the intervention, endodontic treatment, irrigation or disinfection procedures, and relevant clinical outcomes. The principal search concepts included “GentleWave,” “Gentle Wave,” “multisonic ultracleaning,” “multisonic irrigation,” “root canal irrigation,” “endodontic disinfection,” “conventional irrigation,” “syringe irrigation,” “ultrasonic irrigation,” “postoperative pain,” “clinical healing,” and “success.” Boolean operators were used to combine the search concepts. Complete database-specific strategies, including the exact search strings, Boolean operators, field restrictions, and applicable limits, are provided in the Appendices. Reference lists of eligible publications and relevant review articles were also screened manually to identify additional reports that may not have been retrieved through the electronic searches.

Study Selection

All retrieved records were imported into the Elicit systematic review tool (Elicit Research, Oakland, USA), and duplicate records were removed before screening. Two reviewers (TN and AJ) independently screened the titles and abstracts against the predefined eligibility criteria. Full texts were obtained for records considered potentially eligible by either reviewer and for records whose eligibility could not be determined from the title and abstract alone. The same reviewers independently assessed the full-text reports. Disagreements were resolved through discussion and consensus, and consultation with a third reviewer (RR) when required. Reasons for excluding reports after full-text assessment were recorded. 

The publication or report was considered the unit of inclusion, while the underlying participant cohort was considered when determining the independence of the evidence. Possible cohort overlap was assessed by comparing author groups, participating centers, recruitment periods, eligibility criteria, baseline characteristics, sample sizes, intervention protocols, and follow-up periods. Reports were considered definitely or potentially overlapping when several of these characteristics corresponded. Participant numbers from such publications were not summed, and their findings were not interpreted as independent replication.

Data Extraction

Data were extracted independently by two reviewers using a structured and piloted data-extraction form. Disagreements were resolved through discussion and consultation with a third reviewer when necessary. Extracted study characteristics included authors, publication year, country, clinical setting, study design, sample size, number of participants analyzed, participant age, pulpal and periapical diagnoses, tooth type, and follow-up duration. Publication type, funding sources, reported conflicts of interest, and possible relationships with other included publications were also recorded.

Procedural data included the instrumentation system, final apical preparation size and taper, irrigant type and concentration, and irrigation sequence and duration. GentleWave-specific information included the solution sequence, treatment duration, irrigant volume or flow rate, and other reported operating parameters. For comparator groups, the irrigation approach, activation method, needle design, insertion depth, activation duration, and ultrasonic or sonic device parameters were extracted when reported.

Outcome data included the postoperative pain scale, assessment time points, group-level means, measures of dispersion, number of participants reporting pain, analgesic consumption, mechanical allodynia, statistical methods, effect estimates, confidence intervals, and p-values. For clinical and radiographic healing, the outcome definitions, numerator, denominator, follow-up period, and reported healing or success proportion were extracted. The original investigators’ classifications of “healed,” “healing,” “diseased,” or “successful” were retained. Data from different follow-up periods were extracted separately.

Outcome Definitions and Evidence Streams

Two evidence streams were defined before synthesis. The primary comparative evidence stream addressed whether GentleWave improved postoperative pain or other clinical outcomes compared with conventional irrigation. Only studies with a concurrent comparator contributed to conclusions regarding comparative effectiveness. The secondary descriptive evidence stream examined postoperative pain and clinical or radiographic healing reported after GentleWave treatment in prospective single-arm clinical studies. These findings were summarized as outcomes observed following GentleWave treatment but were not interpreted as evidence of superiority, equivalence, or comparative effectiveness. Comparative and noncomparative evidence was therefore presented separately throughout the synthesis.

Risk of Bias Assessment

Risk of bias was assessed independently by two reviewers, with disagreements resolved through discussion and third-reviewer input when required. Randomized controlled trials were evaluated using the Cochrane Risk of Bias 2 (RoB 2) tool [20]. The assessed domains included the randomization process, deviations from intended interventions, missing outcome data, outcome measurement, and selection of the reported result. Domain-level judgments were used to determine the overall risk of bias.

Prospective nonrandomized studies were evaluated using the Risk Of Bias In Non-randomized Studies of Interventions (ROBINS-I) tool [21]. The assessed domains included bias due to confounding, participant selection, intervention classification, deviations from intended interventions, missing data, outcome measurement, and selection of the reported result. The patient-reported nature of postoperative pain was not considered, by itself, to indicate a risk of bias. Judgments instead considered blinding, consistency of outcome assessment, completeness of follow-up, and selective reporting. Domain-level and overall judgments were reported separately for each included publication.

Data Synthesis

A meta-analysis was considered but was not performed because the available reports were insufficiently comparable for a clinically meaningful pooled estimate. Clinical heterogeneity arose from differences in tooth type, pulpal and periapical diagnoses, instrumentation systems, final apical preparation sizes, irrigant concentrations, treatment protocols, and use of ultrasonic activation. Methodological heterogeneity included differences between randomized and nonrandomized designs, comparative and single-arm investigations, outcome definitions, and follow-up periods. Statistical heterogeneity could not be estimated reliably because only two randomized trials contributed comparative postoperative pain data, whereas the remaining evidence was noncomparative or potentially derived from overlapping cohorts.

A structured narrative synthesis was therefore performed. Studies were first grouped into comparative and noncomparative evidence streams and were subsequently organized according to outcome and follow-up period. Comparative postoperative pain findings were summarized using the number of participants analyzed, group means, measures of dispersion, mean differences, 95% confidence intervals, and reported p-values. Where group means and measures of dispersion were available, but a between-group estimate was not reported, the unadjusted mean difference was calculated as the GentleWave mean minus the comparator mean, together with a corresponding 95% confidence interval. Estimates calculated by the review authors were identified accordingly.

Pain scores measured using different instruments or at non-equivalent assessment points were not pooled. Findings from single-arm studies were summarized using numerators, denominators, proportions, and follow-up periods. Healing outcomes from definitely or potentially overlapping publications were reported separately and were not combined into an overall healing estimate. Study design, risk of bias, presence or absence of a comparator, and possible cohort overlap were considered when interpreting each body of evidence.

Certainty of Evidence

The certainty of evidence was assessed separately for each prespecified outcome using the Grading of Recommendations Assessment, Development and Evaluation (GRADE) approach [22]. Randomized evidence initially received a high-certainty rating, whereas nonrandomized evidence initially received a low-certainty rating. Certainty could be downgraded for risk of bias, inconsistency, indirectness, imprecision, or publication bias. The GRADE criteria for upgrading nonrandomized evidence were also considered.

Comparative postoperative pain and noncomparative clinical or radiographic healing were evaluated as separate bodies of evidence. Imprecision was assessed according to the number of participants and events, width of the confidence intervals, inclusion of clinically important benefit or harm within those intervals, and adequacy of the available information relative to an optimal information size. The absence of a pooled estimate was not independently used as a reason for downgrading. Outcomes for which no eligible clinical evidence was available were classified as not estimable. Certainty judgments were made independently by two reviewers, with disagreements resolved through discussion and consensus.

Protocol Deviations

No substantive deviations from the registered protocol were made. Before synthesis, the eligible evidence was organized into primary comparative and secondary descriptive streams to prevent findings from prospective single-arm studies from being interpreted as estimates of comparative effectiveness. This represented a clarification of the synthesis framework rather than a change to the prespecified eligibility criteria.

Results

Study Selection

The searches identified 609 records: PubMed/MEDLINE (n=4), CENTRAL (n=4), Scopus (n=32), Embase (n=87), Web of Science (n=82), Google Scholar (n=200), and EBSCOhost (n=200). After removing 211 duplicates, 398 records underwent title and abstract screening, of which 377 were excluded. Twenty-one reports were sought for retrieval, but four full texts could not be obtained. Of the 17 full-text reports assessed, 12 were excluded because of irrelevant outcomes (n=4), ineligible study populations or designs (n=5), or inadequate or ambiguous data (n=3). Five publications were included in the final synthesis (Figure 1) [23-27].

**Figure 1:**
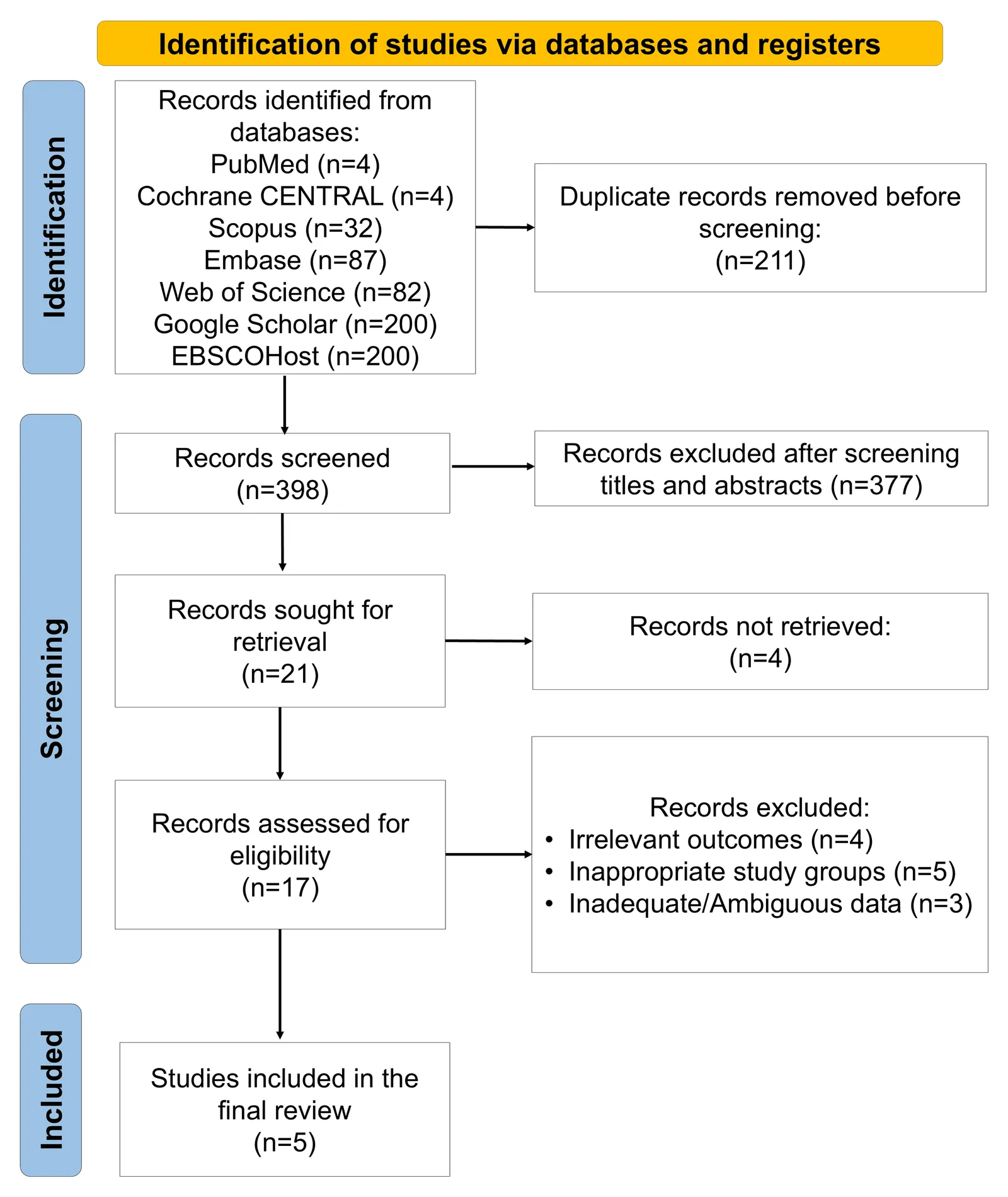
PRISMA flow diagram showing the study selection process in the present systematic review PRISMA: Preferred Reporting Items for Systematic Reviews and Meta-Analyses

Characteristics of the Included Evidence

The five eligible publications comprised two randomized controlled trials evaluating GentleWave against conventional syringe irrigation with ultrasonic activation [26,27] and three prospective noncomparative publications evaluating postoperative pain and healing after GentleWave treatment [23-25]. All investigations were conducted in the United States and included posterior permanent teeth. The principal study and participant characteristics are presented in Table 2.

**Table 2:** Characteristics of the included clinical publications PAI: Periapical Index score GentleWave® system by Sonendo Inc, Laguna Hills, USA.

Publication	Country	Design and evidence stream	Participants analyzed	Age	Teeth and clinical characteristics	Follow-up and cohort relationship
Sigurdsson et al. (2016) [23]	USA	Prospective multicenter, single-arm study; secondary descriptive evidence	89 enrolled; 75 evaluated at 12 months	18-75 years	First and second permanent molars; primary root canal treatment	12 months; same underlying PURE cohort as the six-month publication [24]
Sigurdsson et al. (2016) [24]	USA	Prospective multicenter, single-arm study; secondary descriptive evidence	89 enrolled; 77 evaluated at six months	18-75 years	First and second permanent molars; primary root canal treatment	Six months; same underlying PURE cohort as the 12-month publication [23]
Sigurdsson et al. (2018) [25]	USA	Secondary analysis of two prospective multicenter, single-arm clinical investigations; secondary descriptive evidence	45 enrolled; 44 evaluated at 12 months	Mean 37 ± 11 years; range 19-68 years	First and second permanent molars with baseline PAI ≥3	12 months; possible overlap with participants from related GentleWave clinical investigations
Grigsby et al. (2020) [26]	USA	Single-blind randomized controlled trial; primary comparative evidence	44 randomized; 36 analyzed (18 per group)	24-81 years	Permanent molars and premolars undergoing primary root canal treatment	Postoperative pain assessed through 168 hours
Dale (2021) [27]	USA	Single-blind randomized controlled trial; master’s thesis; primary comparative evidence	43 recruited; 40 analyzed (20 per group)	18-84 years	Symptomatic permanent molars and premolars undergoing primary root canal treatment	Postoperative pain assessed through 168 hours; mechanical allodynia reassessed at the second visit

The two Sigurdsson et al. publications from 2016 reported different follow-up periods for the same cohort of 89 participants: 77 were evaluated at six months [24], and 75 were evaluated at 12 months [23]. The 2018 publication was a secondary analysis of 45 participants with a baseline Periapical Index (PAI) score of at least 3 who were selected from two prospective clinical investigations [25]. Because possible participant overlap between the 2018 analysis and the earlier cohorts could not be excluded, report-level sample sizes were not combined to calculate a total number of unique participants.

Grigsby et al. randomized 44 participants, of whom 36 returned the postoperative pain assessments and were included in the analysis [26]. Dale recruited 43 participants, with 40 completing follow-up and contributing outcome data, comprising 20 participants in each treatment group [27].

Instrumentation and Irrigation Protocols

The instrumentation and irrigation protocols are summarized in Table 3. In the three prospective noncomparative publications, canals were prepared to approximately ISO 20, generally using hand K-files followed by ProTaper® F1 (#20/.07) (Dentsply Sirona Inc., Charlotte, USA). The GentleWave treatment sequence used sodium hypochlorite at a concentration of 3% or lower, water, 8% EDTA for two minutes, and a final water rinse [23-25].

**Table 3:** Instrumentation and irrigation protocols EDTA - ethylenediaminetetraacetic acid; ISO - International Organization for Standardization GentleWave® by Sonendo Inc., Laguna Hills, USA. ProTaper by Dentsply Sirona Inc., Charlotte, USA.

Publication	Mechanical preparation	GentleWave® irrigation protocol	Comparator protocol
Sigurdsson et al. (2016) [23]	Hand K-files #10-15 followed by ProTaper® F1 (#20/.07); final preparation approximately ISO 20	Sodium hypochlorite ≤3%, water, 8% EDTA for two minutes, and final water rinse; sealed handpiece with suction-assisted evacuation	None
Sigurdsson et al. (2016) [24]	Hand K-files #10-15 followed by ProTaper F1 (#20/.07); final preparation approximately ISO 20	Sodium hypochlorite ≤3%, water, 8% EDTA for two minutes, and final water rinse; sealed handpiece with suction-assisted evacuation	None
Sigurdsson et al. (2018) [25]	Hand K-files #10-15 followed by ProTaper F1 (#20/.07); final preparation approximately ISO 20	Approximately 3% sodium hypochlorite, water, 8% EDTA for two minutes, and final water rinse	None
Grigsby et al. (2020) [26]	GentleWave: minimum #20/.04 or .06; control: minimum #25/.04; final size determined according to canal anatomy	Sodium hypochlorite used during instrumentation followed by the manufacturer-selected GentleWave cycle; reported flow approximately 50 ± 10 mL/min for molars and 35 ± 10 mL/min for premolars	Side-vented syringe irrigation with 5.25% sodium hypochlorite; ultrasonic activation for 30 seconds per canal; 3 mL of 17% EDTA for one minute; final sodium hypochlorite and conditional ethanol rinse
Dale (2021) [27]	First visit" GentleWave: maximum #20/.04; control: minimum #25/.04	Sodium hypochlorite during instrumentation followed by the GentleWave treatment cycle; calcium hydroxide placed between visits	First visit: side-vented syringe with 5.25% sodium hypochlorite and ultrasonic activation for 30 seconds per canal; second visit: completion of instrumentation, 17% EDTA for one minute, sodium hypochlorite, and a conditional 95% ethanol rinse

In the randomized trial by Grigsby et al., GentleWave canals were prepared to a minimum of #20/.04 or .06, whereas comparator canals were prepared to at least #25/.04 [26]. The comparator involved 5.25% sodium hypochlorite during instrumentation, ultrasonic activation for 30 seconds per canal, 17% EDTA for one minute, and a final rinse determined by the sealer used. Dale similarly used a maximum preparation of #20/.04 in the GentleWave group and a minimum of #25/.04 in the conventional group during the first treatment visit [27]. This trial used a two-visit protocol, with calcium hydroxide placed between visits and conventional completion of instrumentation and irrigation at the second visit.

Comparative Postoperative Pain

The numerical pain, mechanical allodynia, and healing outcomes are presented in Table 4. Both randomized trials used a 0-100 numeric rating scale and assessed postoperative pain at six, 24, 72, and 168 hours [26,27]. In the trial by Grigsby et al., 15 of 18 GentleWave participants and 13 of 18 control participants reported at least one episode of postoperative pain, corresponding to a risk ratio of 1.15 [26]. Mean pain scores were lower in the GentleWave group at every postoperative assessment. The calculated mean difference was −11.66 points at six hours, −11.55 points at 24 hours, −8.11 points at 72 hours, and −2.44 points at 168 hours. All confidence intervals included the null value. Pain decreased significantly over time in both groups.

**Table 4:** Clinical outcomes reported in the included publications NRS: numeric rating scale; RR: risk ratio; MD: mean difference GentleWave® by Sonendo Inc., Laguna Hills, USA.

Publication and evidence stream	Postoperative pain or mechanical allodynia	Clinical and radiographic healing	Comparative effect estimate
Sigurdsson et al. (2016), 12-month report [23]	Moderate pain reported by 3.8% within two days; no pain at 14 days or later assessments	69 healed, four healing, and two diseased; combined success 73/75 (97.3%; 95% CI, 90.8%-99.3%) at 12 months	No comparator
Sigurdsson et al. (2016), six-month report [24]	Approximately 3% reported moderate pain within two days; no severe pain	60 healed, 15 healing, and two diseased; combined success 75/77 (97.4%; 95% CI, 91.0%-99.3%) at six months	No comparator
Sigurdsson et al. (2018) [25]	Mild pain in 7/45 participants at two days; no moderate or severe pain at two, seven, or 14 days	36 healed, seven healing, and one diseased; combined success 43/44 (97.7%; 95% CI, 88.2%-99.6%) at 12 months	No comparator
Grigsby et al. (2020) [26]	Any pain: GentleWave 15/18 versus control 13/18. Mean NRS, GentleWave versus control: six hours, 11.56 ± 9.94 versus 23.22 ± 25.38; 24 hours, 7.78 ± 10.88 versus 19.33 ± 21.46; 72 hours, 5.00 ± 7.28 versus 13.11 ± 18.56; 168 hours, 2.78 ± 7.32 versus 5.22 ± 10.46	Not assessed	Any pain: RR 1.15 (95% CI, 0.81-1.64). MDs: six hours, −11.66 (−24.98 to 1.66); 24 hours, −11.55 (−23.23 to 0.13); 72 hours, −8.11 (−17.85 to 1.63); 168 hours, −2.44 (−8.58 to 3.70)
Dale (2021) [27]	Mean NRS, GentleWave versus control: six hours, 24 (15-33) versus 22 (13-30); 24 hours, 21 (12-30) versus 25 (16-34); 72 hours, 15 (6-23) versus 17 (9-26); 168 hours, 6 (0-14) versus 8 (0-16). Bite-force increase: 96 MPa (23-169) versus 118 MPa (45-191)	Not assessed	MDs: six hours, 2 (approximate 95% CI, −10.0 to 14.0); 24 hours, −4 (−16.3 to 8.3); 72 hours, −2 (−13.6 to 9.6); 168 hours, −2 (−12.3 to 8.3). Between-group bite-force comparison: p=0.67

In the trial by Dale, mean pain differences between GentleWave and conventional treatment were 2 points at six hours, −4 points at 24 hours, −2 points at 72 hours, and −2 points at 168 hours [27]. The confidence intervals included both possible benefit and no difference. The prevalence of pain of any intensity was also similar between groups. At six hours, pain was reported by 85% of GentleWave participants and 75% of controls. The corresponding proportions were 65% and 80% at 24 hours, 55% in both groups at 72 hours, and 40% in both groups at 168 hours. Dale additionally assessed mechanical allodynia using changes in bite-force thresholds. Bite force increased by a mean of 118 MPa in the conventional group and 96 MPa in the GentleWave group. The between-group difference was not statistically significant (p=0.67) [27].

Descriptive Postoperative Pain

The three prospective noncomparative publications reported limited early postoperative pain after GentleWave treatment [23-25]. In the six-month publication, approximately 3% of participants reported moderate pain within two days, and no severe pain was reported [24]. In the 12-month publication from the same cohort, 3.8% reported moderate pain within two days, with no pain reported at 14 days or subsequent follow-up assessments [23]. In the 2018 secondary analysis, seven of 45 participants reported mild pain at two days, while no moderate or severe pain was reported at two, seven, or 14 days [25]. These findings arose from related or potentially overlapping participant populations and were therefore not treated as independent replication.

Clinical and Radiographic Healing

Clinical and radiographic healing was reported only in the three prospective noncomparative publications [23-25]. Outcomes were evaluated using clinical findings and PAI-based radiographic classifications. Neither randomized trial reported long-term healing. At six months, 60 teeth were classified as healed, 15 as healing, and two as diseased, resulting in a combined healing or success proportion of 75/77 (97.4%) [24]. At 12 months, 69 teeth were healed, four were healing, and two remained diseased, corresponding to 73/75 (97.3%) in the same underlying cohort [23]. In the 2018 analysis of teeth with baseline PAI scores of at least 3, 36 were healed, seven were healing, and one was diseased, corresponding to 43/44 (97.7%) at 12 months [25]. Because these publications lacked concurrent comparator groups and potentially included overlapping participants, the proportions were reported separately and were not combined.

Clinical Disinfection-Related Outcomes

None of the eligible clinical publications reported quantitative bacterial reduction, smear-layer removal, or irrigant penetration into dentinal tubules in a form suitable for comparative clinical synthesis [23-27]. Consequently, the clinical evidence for these outcomes was unavailable rather than indicative of either effectiveness or ineffectiveness.

Risk of Bias

Both randomized trials were judged to raise some concerns overall using the Risk of Bias 2 (RoB 2) tool by the Cochrane Collaboration, London, UK (Figure 2) [20]. Grigsby et al. adequately generated the randomization sequence and used a consistent outcome measure; however, concerns arose from post-randomization exclusions, incomplete pain assessments in eight of 44 randomized participants, and the absence of an accessible prespecified analysis plan [26]. Dale had limited reporting of sequence generation and allocation concealment, although missing data were limited, outcome measurement was appropriate, and the study was prospectively registered [27].

**Figure 2:**
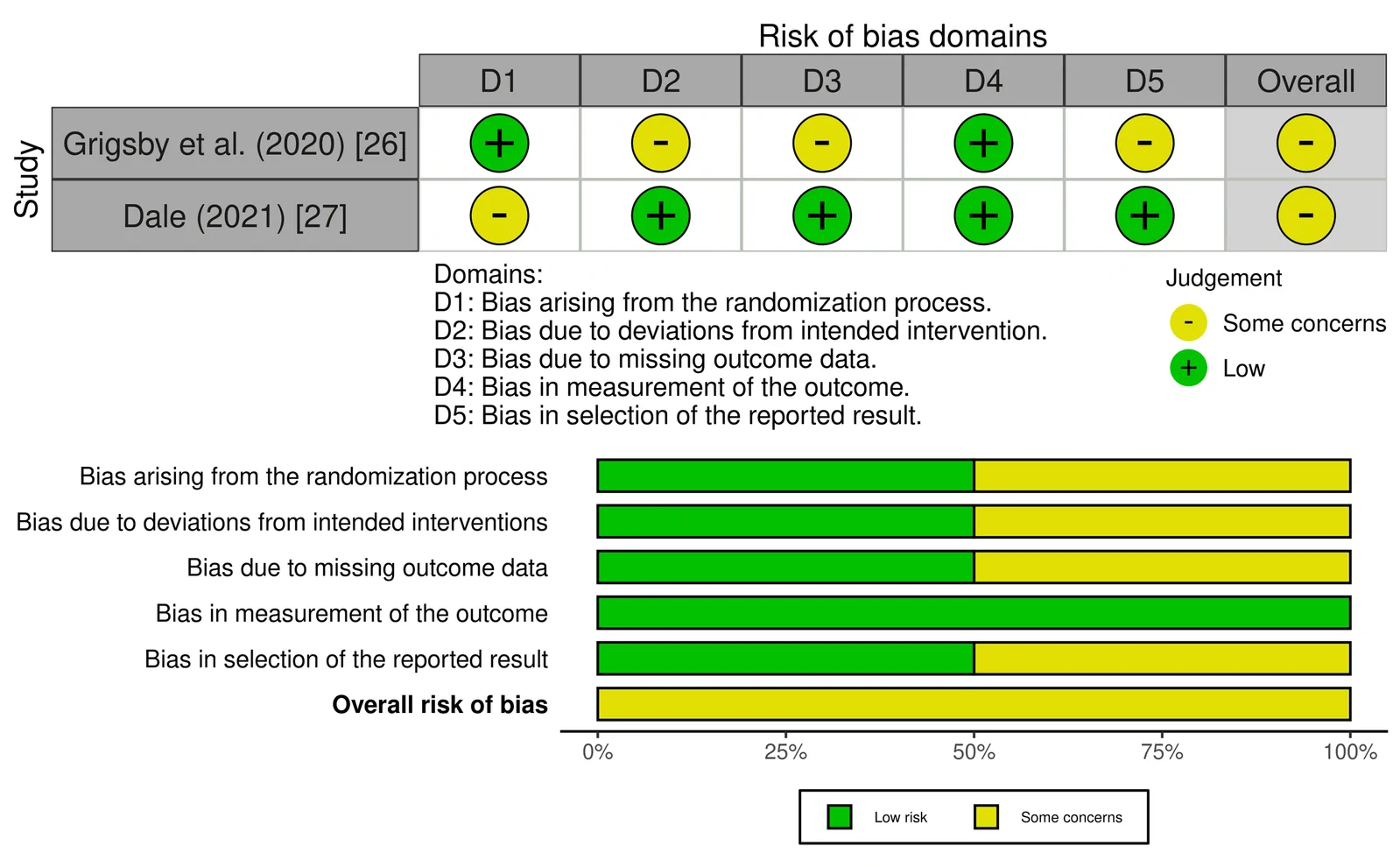
Risk of bias across the included randomized controlled trials usign the Cochrane RoB 2 tool Risk of Bias 2 (RoB 2) tool by the Cochrane Collaboration, London, UK [20].

Postoperative pain is inherently a patient-reported outcome, and its subjective nature was not considered a source of bias by itself because pain intensity was appropriately assessed using a standardized numerical rating scale. Risk of bias judgments instead considered the consistency of outcome measurement, missing outcome data, deviations from the intended interventions, and the possibility of selective reporting.

Using ROBINS-I, the six- and 12-month publications from the prospective PURE cohort were judged to have a moderate overall risk of bias, principally because of the absence of a comparator, potential confounding, incomplete follow-up, and limitations in outcome reporting (Figure 3) [23,24]. The 2018 secondary analysis was judged to have a serious overall risk of bias because participants with larger baseline periapical lesions were selected from two earlier clinical investigations, creating additional concerns regarding participant selection, confounding, and selective analysis [25].

**Figure 3:**
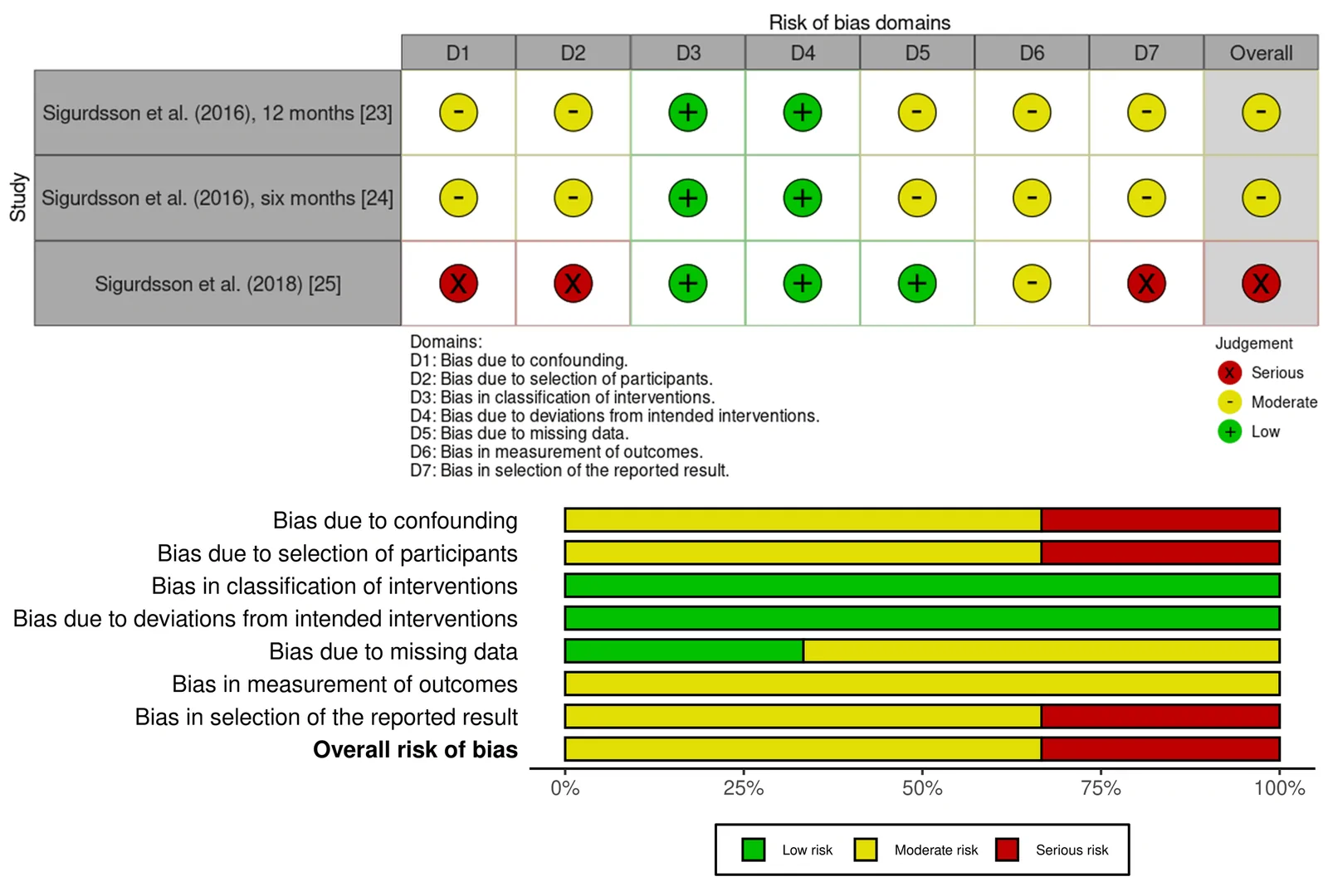
Risk of bias across the included non-randomized clinical studies using the ROBINS-I tool Risk Of Bias In Non-randomized Studies of Interventions (ROBINS-I) by the Cochrane Collaboration, London, UK [21].

Certainty of Evidence

The GRADE assessment is presented in Table 5. Comparative evidence for postoperative pain was rated as low certainty. The evidence was downgraded for risk of bias because both randomized trials raised some concerns and for imprecision because only 76 participants were analyzed and the confidence intervals included possible benefit, no difference, and, at some time points, possible harm.

**Table 5:** GRADE certainty of evidence for prespecified clinical outcomes Randomized evidence initially received a high-certainty rating, whereas nonrandomized evidence initially received a low-certainty rating. The healing evidence reached the lowest GRADE category after downgrading and therefore remained classified as very low certainty. No upgrading criteria were identified. GentleWave by Sonendo Inc., Laguna Hills, USA. GRADE: Grading of Recommendations Assessment Development and Evaluation; RoB 2: Risk of Bias 2 tool by the Cochrane Collective, London, UK.

Outcome	Evidence and principal finding	Risk of bias	Inconsistency	Indirectness	Imprecision	Publication bias	Overall certainty
Comparative postoperative pain	Two randomized controlled trials; 76 participants analyzed. No clear between-group difference was demonstrated from 6 to 168 hours.	Serious; downgraded one level because both trials raised some concerns in at least one RoB 2 domain.	Not serious; the trials did not demonstrate a consistent advantage for either intervention.	Not serious; both trials directly compared GentleWave with conventional syringe irrigation combined with ultrasonic activation.	Serious; downgraded one level because of the small sample and confidence intervals that included possible benefit, no difference, and possible harm.	Not assessable because only two trials were available; no downgrade applied.	Low
Clinical and radiographic healing	Three prospective noncomparative publications reported healing or success proportions of 97.3% to 97.7% at 6 to 12 months. The number of unique participants was uncertain.	Serious; downgraded one level because of confounding, participant selection, incomplete follow-up, outcome measurement limitations, and the absence of concurrent controls.	Not serious; the reported proportions were similar, although two publications represented the same cohort and the third may have included overlapping participants.	Serious; downgraded one level because single-arm proportions did not directly estimate healing relative to conventional irrigation.	Serious; downgraded one level because the number of unique participants was uncertain and the confidence intervals remained wide.	Not assessable because only a few related publications were available; no separate downgrade applied.	Very low
Quantitative clinical disinfection outcomes	No eligible clinical publication reported bacterial reduction, smear-layer removal, or irrigant penetration data suitable for synthesis.	Not assessed	Not assessed	Not assessed	Not assessed	Not assessed	Not estimable

The certainty of evidence for clinical and radiographic healing was rated as very low. The evidence was limited to three noncomparative publications, two of which reported different follow-up periods for the same cohort, while the third may have included overlapping participants. Additional limitations included risk of bias, absence of concurrent controls, uncertain unique sample size, and limited precision. Certainty for quantitative clinical disinfection outcomes was not estimable because no eligible publication reported data suitable for synthesis.

Discussion

This systematic review evaluated the clinical evidence for the GentleWave system during primary nonsurgical root canal treatment. Five eligible publications were identified, comprising two randomized comparative trials and three prospective noncomparative publications [23-27]. The randomized evidence addressed short-term postoperative pain, whereas healing was reported only in single-arm investigations. Furthermore, two publications reported different follow-up periods for the same cohort, and possible overlap with the 2018 secondary analysis could not be excluded. The number of publications should therefore not be interpreted as the number of independent studies or participant populations.

The two randomized trials did not demonstrate a clear difference in postoperative pain between GentleWave and conventional syringe irrigation combined with ultrasonic activation [26,27]. Pain intensity decreased during the first postoperative week in both groups. The calculated mean differences favored GentleWave at several time points in the Grigsby et al. trial, particularly at six and 24 hours, but the confidence intervals included no difference [26]. In the Dale trial, the between-group differences were small, inconsistent in direction, and accompanied by wide confidence intervals [27]. Taken together, these findings do not establish a clinically meaningful short-term pain advantage for either treatment approach.

The absence of statistically significant differences should not be interpreted as evidence of equivalence. Only 76 participants contributed to the randomized pain analysis, and neither trial was designed as an equivalence or noninferiority study. The confidence intervals remained compatible with possible benefit, no difference, and, at some time points, possible harm. Consequently, the randomized evidence indicates uncertainty regarding the magnitude of any difference rather than demonstrating that the two approaches produce identical outcomes.

Postoperative pain was evaluated using patient-reported numeric rating scales. Pain is inherently subjective, but patient self-report is the appropriate method for measuring this outcome and should not itself be considered a source of bias. The more relevant methodological considerations are whether participants were blinded, whether the same instrument and assessment schedule were used in both groups, whether missing responses were balanced, and whether analyses followed a prespecified plan. Both trials used the same pain scale and assessment points within their respective comparisons, although incomplete follow-up and limited reporting of randomization or analysis procedures resulted in some risk-of-bias concerns [26,27].

The randomized comparisons also differed in several procedural aspects that complicate attribution of outcomes specifically to the irrigation system. GentleWave canals generally underwent smaller apical preparations, whereas comparator canals were prepared to larger minimum sizes. Differences were also present in irrigant concentrations, activation procedures, final rinses, treatment visits, and interappointment medication [26,27]. Dale used a two-visit design with calcium hydroxide placement, while Grigsby evaluated treatment completed within a shorter clinical sequence. Because apical preparation and irrigant concentration can influence microbial reduction and irrigant exchange, these protocol differences may have affected clinical outcomes independently of the irrigation device [28].

Several mechanisms have been proposed to explain how GentleWave may improve irrigant movement while permitting conservative canal preparation. These include multisonic energy, continuous fluid exchange, and suction-assisted evacuation under negative pressure [15,16]. Laboratory investigations have reported effects on microbial products within experimentally infected canals [17,18]. However, mechanistic plausibility and laboratory performance cannot be assumed to establish superior clinical effectiveness. The present clinical evidence supports conclusions principally about short-term postoperative pain and does not confirm that the proposed hydrodynamic or acoustic mechanisms translate into improved disinfection or long-term treatment success.

The three prospective noncomparative publications reported combined healing or success proportions of approximately 97% at six to 12 months [23-25]. These proportions appear favorable but require cautious interpretation. The six- and 12-month publications described follow-up of the same initial cohort rather than independent replication [23,24]. The 2018 report was a secondary analysis of participants with larger baseline periapical lesions drawn from two prospective investigations, and its independence from the earlier participant population could not be fully established [25]. The three percentages therefore should not be combined or interpreted as consistent findings from three independent clinical studies.

More importantly, none of the healing publications included a concurrent conventional-irrigation group. Improvement in clinical and radiographic findings after root canal treatment may be influenced by case selection, baseline lesion characteristics, instrumentation, obturation, coronal restoration, operator experience, and host-related factors. In the absence of a comparator, the observed healing cannot be attributed specifically to GentleWave. The randomized trials assessed outcomes only during the first postoperative week and did not provide comparative radiographic healing data [26,27]. Consequently, the available evidence cannot determine whether GentleWave improves, reduces, or produces similar long-term healing compared with conventional activated irrigation.

The healing evidence was additionally limited by moderate-to-serious risk of bias, uncertain cohort independence, and commercial involvement. The 2016 PURE investigation was funded by Sonendo, while the 2018 investigation received partial company funding, and consultancy relationships were disclosed by some investigators [23-25]. Disclosure does not demonstrate that the reported findings were invalid, but the concentration of the healing evidence within closely related and commercially supported investigations limits independent replication. These factors contributed to the very-low-certainty rating for healing.

No eligible clinical publication reported quantitative bacterial reduction, smear-layer scores, or the depth of irrigant penetration into dentinal tubules in a form suitable for comparative synthesis [23-27]. This absence should be interpreted as an evidence gap rather than evidence that GentleWave is either effective or ineffective for intracanal disinfection. Laboratory endpoints can provide mechanistic information, but clinical sampling studies are needed to determine whether observed differences in cleaning translate into relevant biological or patient-centered benefits.

From a clinical perspective, the current evidence does not establish the superiority or equivalence of GentleWave over contemporary conventional irrigation with ultrasonic activation. The randomized findings suggest that GentleWave can be used without an evident increase in mean short-term pain, but they do not demonstrate a consistent analgesic advantage. Likewise, the high healing proportions from single-arm studies are insufficient to support comparative claims. Decisions regarding adoption should therefore consider equipment cost, operator training, case selection, accessibility, and the effectiveness of established irrigation protocols, rather than assuming improved clinical outcomes from the proposed mechanism alone.

This review has several strengths. It followed a registered protocol, used independent study selection and data extraction, and assessed risk of bias using design-appropriate tools. Comparative and noncomparative evidence were separated to avoid interpreting single-arm healing proportions as comparative effects. Individual randomized trial effects were presented with confidence intervals rather than relying only on p-values, and potentially overlapping publications were identified instead of combining their participant numbers. Certainty was also assessed separately for postoperative pain, healing, and clinical disinfection outcomes.

The underlying evidence nevertheless had important limitations. All investigations were conducted in the United States, sample sizes were modest, and only two trials provided direct comparisons. Instrumentation sizes, irrigant concentrations, treatment schedules, comparator procedures, and follow-up periods varied. The randomized trials provided only short-term pain outcomes, while the healing publications lacked concurrent controls. The independence of the prospective cohorts was uncertain, objective clinical disinfection outcomes were absent, and much of the healing evidence came from related investigators with similar institutional affiliations. These factors restrict both causal interpretation and generalizability.

The review process also had limitations. Restricting inclusion to English-language full texts may have introduced language bias, while the small number of publications prevented formal assessment of publication bias. Google Scholar uses dynamic relevance-based rankings, and screening was limited to the first 200 results; therefore, the search may not be fully reproducible, and eligible lower-ranked records may have been missed. Although the individual databases accessed through EBSCOhost were reported, database availability and coverage may vary according to institutional subscriptions. No dedicated dissertation database was searched, and relevant theses were identified through Google Scholar. In addition, four reports sought for full-text retrieval could not be obtained. Studies published after the final search date of October 21, 2025, were also outside the scope of this review. These limitations may have resulted in incomplete retrieval of relevant published or unpublished evidence.

Future research should include independently conducted, adequately powered, multicenter randomized trials comparing GentleWave with clearly standardized contemporary irrigation protocols. Intervention groups should use comparable instrumentation dimensions, irrigant concentrations, treatment visits, and obturation procedures wherever clinically feasible. Trials should report prespecified effect estimates with 95% confidence intervals and should include short-term pain, adverse events, microbiological reduction, and blinded clinical and radiographic healing assessments extending for at least 12 to 24 months. Such studies are necessary to determine whether the proposed mechanistic advantages of GentleWave provide clinically meaningful benefits beyond those achieved with established activated irrigation techniques.

## Conclusions

GentleWave-assisted root canal treatment did not demonstrate a clear advantage over conventional syringe irrigation combined with ultrasonic activation for short-term postoperative pain, although the available comparative evidence was limited and of low certainty. High healing proportions were reported in prospective single-arm publications, but these findings represented very-low-certainty evidence because of the absence of concurrent controls, risk of bias, and definite or possible cohort overlap. Clinical evidence concerning bacterial reduction, smear-layer removal, and irrigant penetration was unavailable; therefore, neither superiority nor equivalence regarding disinfection or long-term healing can be concluded. Adequately powered, independent randomized trials with standardized protocols and long-term clinical, radiographic, and microbiological outcomes are required.

## Appendices

**Table 6:** Database-specific electronic search strategies

Database/platform	Search strategy	Coverage and limits
PubMed/MEDLINE	(("GentleWave"[Title/Abstract] OR "Gentle Wave"[Title/Abstract] OR "multisonic ultracleaning"[Title/Abstract] OR "multisonic irrigation"[Title/Abstract]) AND ("Root Canal Irrigation"[MeSH Terms] OR "Root Canal Therapy"[MeSH Terms] OR endodontic*[Title/Abstract] OR "root canal"[Title/Abstract]) AND (irrigat*[Title/Abstract] OR disinfect*[Title/Abstract] OR debrid*[Title/Abstract] OR clinical[Title/Abstract] OR pain[Title/Abstract] OR heal*[Title/Abstract] OR success[Title/Abstract] OR bacter*[Title/Abstract] OR "smear layer"[Title/Abstract] OR dentin*[Title/Abstract]))	From database inception through 21 October 2025; no publication-year filter.
Scopus and Embase	TITLE-ABS-KEY(("GentleWave" OR "Gentle Wave" OR "multisonic ultracleaning" OR "multisonic irrigation") AND (endodontic* OR "root canal") AND (irrigat* OR disinfect* OR debrid* OR clinical OR pain OR heal* OR success OR bacter* OR "smear layer" OR dentin*))	From database inception through 21 October 2025; document type and language assessed during screening.
Web of Science Core Collection	TS=(("GentleWave" OR "Gentle Wave" OR "multisonic ultracleaning" OR "multisonic irrigation") AND (endodontic* OR "root canal") AND (irrigat* OR disinfect* OR debrid* OR clinical OR pain OR heal* OR success OR bacter* OR "smear layer" OR dentin*))	From database inception through 21 October 2025; all available citation indexes searched.
Cochrane CENTRAL	#1 MeSH descriptor: [Root Canal Irrigation] explode all trees #2 MeSH descriptor: [Root Canal Therapy] explode all trees #3 (GentleWave OR "Gentle Wave" OR "multisonic ultracleaning" OR "multisonic irrigation"):ti,ab,kw #4 (endodontic* OR "root canal"):ti,ab,kw #5 (irrigat* OR disinfect* OR debrid* OR clinical OR pain OR heal* OR success OR bacter* OR "smear layer" OR dentin*):ti,ab,kw #6 #3 AND (#1 OR #2 OR #4) AND #5	From database inception through 21 October 2025; trials and other prospectively registered clinical reports considered.
Google Scholar	("GentleWave" OR "Gentle Wave" OR "multisonic ultracleaning" OR "multisonic irrigation") ("root canal" OR endodontic) (irrigation OR disinfection OR debridement OR clinical OR pain OR healing OR success OR bacteria OR "smear layer")	Supplementary search through 21 October 2025; results screened in relevance order.
EBSCOhost platform	TX (GentleWave OR "Gentle Wave" OR "multisonic ultracleaning" OR "multisonic irrigation") AND TX (endodontic* OR "root canal") AND TX (irrigat* OR disinfect* OR debrid* OR clinical OR pain OR heal* OR success OR bacter* OR "smear layer" OR dentin*)	From database inception through 21 October 2025; MEDLINE, MEDLINE Ultimate, MEDLINE with Full Text, CINAHL, CINAHL Ultimate, CINAHL Plus, Biotechnology Source, and Biomedical Reference Collection.
